# Composite quantile regression approach to batch effect correction in microbiome data

**DOI:** 10.3389/fmicb.2025.1484183

**Published:** 2025-02-25

**Authors:** Jiwon Park, Taesung Park

**Affiliations:** ^1^Interdisciplinary Program of Bioinformatics, Seoul National University, Seoul, Republic of Korea; ^2^Department of Statistics, Seoul National University, Seoul, Republic of Korea

**Keywords:** batch effect, composite quantile regression, microbiome, zero inflation, over-dispersion

## Abstract

**Background:**

Batch effects refer to data variations that arise from non-biological factors such as experimental conditions, equipment, and external factors. These effects are considered significant issues in the analysis of biological data since they can compromise data consistency and distort actual biological differences, which can severely skew the results of downstream analyses.

**Method:**

In this study, we introduce a new approach that comprehensively addresses two types of batch effects: “systematic batch effects” which are consistent across all samples in a batch, and “nonsystematic batch effects” which vary depending on the variability of operational taxonomic units (OTUs) within each sample in the same batch. To address systematic batch effects, we apply a negative binomial regression model and correct for consistent batch influences by excluding fixed batch effects. Additionally, to handle nonsystematic batch effects, we employ composite quantile regression. By adjusting the distribution of OTUs to be similar based on a reference batch selected using the Kruskal-Walis test method, we consider the variability at the OTU level.

**Results:**

The performance of the model is evaluated and compared with existing methods using PERMANOVA R-squared values, Principal Coordinates Analysis (PCoA) plots and Average Silhouette Coefficient calculated with diverse distance-based metrics. The model is applied to three real microbiome datasets: Metagenomic urine control data, Human Immunodeficiency Virus Re-analysis Consortium data, and Men and Women Offering Understanding of Throat HPV study data. The results demonstrate that the model effectively corrects for batch effects across all datasets.

## 1 Introduction

The human microbiome, comprising diverse microorganisms across various body sites, is crucial for understanding health and disease dynamics (Ursell et al., 2012; Wang and LêCao, 2020). Advances in sequencing technologies have deepened our understanding by enabling researchers to not only identify microbial species but also to understand their functional roles within the ecosystem. However, these advancements have also highlighted challenges like batch effects—discrepancies across sample batches that can distort biological insights (Leek and Storey, 2007). That is, batch effects refer to systematic and non-systematic discrepancies or variations that arise during the processing of samples in a study (Lazar et al., 2013; Wang and LêCao, 2020). Systematic batch effects represent the consistent differences across all samples within a batch, and nonsystematic batch effects demonstrate variability that is dependent on the diversity of OTUs present within each individual sample of the same batch (Soneson et al., 2014). Key factors contributing to these effects include variations in sample collection, DNA extraction methods, sequencing protocols, and data analysis techniques. Microbiome data's inherent properties—high zero-inflation, over-dispersion—exacerbate the impact of batch effects. Zero-inflation indicates the presence of many zeros in the data, which highlights the scarcity or absence of certain microbial species in many samples. Over-dispersion arises from individual variability and technical differences in sequencing depth, complicating data analysis.

While numerous methods exist for correcting batch effects in high-throughput data, many assume continuous data and are not directly applicable to microbiome data, which typically consist of count data. Traditional methods, such as the ComBat algorithm (Johnson et al., 2007), typically assume a Gaussian distribution. Based on this, extensions for count data such as RNA-seq have been developed that utilize a negative binomial distribution. This approach is more aligned with the inherent properties of the data and effectively adjusts for consistent batch patterns. However, while this method is applicable for addressing systematic batch effects, it struggles to fully correct for non-systematic batch influences caused by irregular experimental errors or the unique characteristics of individual samples.

Another approach, Meta-analysis Methods with a Uniform Pipeline for Heterogeneity in microbiome studies (MMUPHin) provides a comprehensive solution for managing heterogeneity in microbiome studies (Ma et al., 2022). This method includes joint normalization and meta-analysis techniques specifically designed for the unique characteristics of microbiome data. Its adaptability is especially effective for handling the diverse and often non-parametric nature of microbiome count data. However, MMUPHin assumes the data to be Zero-inflated Gaussian, which is primarily suitable only for certain transformations of relative abundance data, such as taxon counts normalized by each sample's library size. This assumption limits its applicability, indicating a need for more flexible approaches in certain scenarios. Percentile normalization is a method by which data can be converted to a uniform distribution based on percentiles (Gibbons et al., 2018). This transformation helps mitigate the effects of over-dispersion and the high zero count. However, a key limitation of percentile-normalization lies in its potential to oversimplify complex data structures, possibly leading to the loss of meaningful biological variance. This issue can be particularly critical when dealing with highly diverse microbiome samples. Recently, new methods for batch effect correction have been introduced, tailored specifically to design unique characteristics of microbiome data. Among those approaches, Conditional Quantile Regression (ConQuR) (Ling et al., 2021a,b) utilizes a conditional quantile regression approach for batch effect correction, enabling the generation of corrected OTUs. ConQuR independently processes each OTU without assuming a specific distribution, offering flexible handling of data. However, when OTUs across batches exhibit significantly different distributions, each quantile may represent different characteristics, potentially compromising the consistency of the analysis results. While using a reference batch to standardize OTU distributions can address fundamental inter-batch differences, the effectiveness of this approach heavily depends on the extent of these differences and the appropriateness of the chosen reference batch. If the reference batch does not adequately represent the characteristics of other batches, this method may introduce additional distortions.

## 2 Materials and methods

### 2.1 Microbiome datasets

#### 2.1.1 Human immunodeficiency virus re-analysis consortium datasets (HIVRC)

We used the first dataset from multiple individual studies derived through the Human Immunodeficiency Virus Re-analysis Consortium (HIVRC) (Deeks et al., 2015; Tuddenham et al., 2020; Ling et al., 2021a,b). This integrated dataset, based on fecal samples, provides microbial profiles indicative of the gut microbiome. It comprises 17 individual datasets, including 16S rRNA gene sequences from both HIV-uninfected (HIV-) and HIV-infected (HIV+) patients. Out of the 17 studies within the HIVRC, we selected 4 specific datasets for our analysis, namely those from Noguera-Julian, Pinto-Cardoso, Serrano-Villar, and Vesterbacka (Table 1). The selection criteria were based on the study design (either case-control or cross-sectional) and the sample type. These criteria ensured that the selected studies were compatible and relevant for our model validation purposes. Publicly available dataset was used in this study.

**Table 1:** Summary of the sample size and covariates in HIVRC datasets.

**Batch ID (study)**	**Sample size**	**HIV status = 1(%)**	**Age [mean(SD)]**	**Gender = 1 (%)**
Noguera-Julian	232	199 (85.8)	42.3 (10.7)	171 (73.7)
Pinto-Cardoso	43	33 (76.7)	39.9 (10.2)	35 (81.4)
Serrano-Villar 2017	23	21 (91.3)	42.4 (8.63)	23 (100)
Vesterbacka	62	47 (75.8)	46.9 (9.61)	31 (50)

The provided HIV status includes the number and proportion of patients diagnosed with HIV. The Age variable presents the mean and standard deviation, while the Gender variable reports the number and percentage of male participants.

#### 2.1.2 Men and women offering understanding of throat HPV study dataset

We used the second dataset from the Men and Women Offering Understanding of Throat Human Papillomavirus (MOUTH) study (Zhang et al., 2022). This cross-sectional study includes 16S rRNA gene sequences from participants with oncogenic oral HPV infection or HPV seropositive antibodies. The dataset is composed of samples distributed across seven distinct sequencing batches, each identified by a unique Batch ID. The HPV dataset includes various covariates, summarized as follows: sample size, age [mean (SD)], smoking status, sexual orientation, and HPV status. Smoking status is categorized as Never smoker (0), Former smoker (1), and Current smoker (2), with the reported percentages indicating the proportion of participants who have ever smoked (including both former and current smokers). Sexual orientation is categorized as Heterosexual (0), Homosexual (1), and Others (2). HPV status represents the percentage of participants with a positive HPV status. This dataset is divided into 7 batches based on the experimental plates used during sequencing, allowing for the consideration of potential batch effects arising from differences in sample processing. These variables, including smoking status and sexual orientation, are incorporated into the analysis to control for potential confounding factors (Table 2). Publicly available dataset was used in this study.

**Table 2:** Summary of the sample size and covariates in HPV datasets.

**Batch ID (plate ID)**	**Sample size**	**Age [mean (SD)]**	**Smoking = True (%)**	**Sexual orientation; Heterosexual = 0, Homosexual = 1, Others = 2**	**HPV status = *p*os (%)**
p68_s01_JH1_16SV4	43	54.6 (10.6)	19 (44.1)	36 (84.7)/3 (7.1)/ 3 (7.1)	3 (6.9)
p68_s02_JH2_16SV4	49	52.8 (9.1)	18 (36.7)	41 (89.1)/1 (2.1)/ 4 (8.6)	3 (6.2)
p68_s03_JH3_16SV4	56	54.3 (9.6)	25 (44.6)	49 (87.5)/3 (5.3)/ 4 (7.1)	2 (3.5)
p68_s04_JH4_16SV4	79	54.1 (9.1)	32 (40.5)	71 (92.2)/4 (5.1)/2 (2.5)	3 (3.7)
p68_s05_JH5_16SV4	89	54 (10.1)	34 (38.2)	83 (93.2)/3 (3.3)/3 (3.3)	2 (2.2)
p68_s06_JH6_16SV4	88	53.3 (10.3)	38 (43.1)	67 (77)/7 (7)/13 (14.9)	20 (23.2)
p68_s07_JH7_16SV4	91	56.7 (11.6)	43 (47.2)	74 (85)/8 (9.1)/5 (5.7)	17 (18.6)

The provided smoking indicator represents the percentage of HIV-positive participants (Pinto-Cardoso et al., 2017). The sexual orientation variable reports the number and proportion of participants based on their sexual orientation (0 = heterosexual, 1 = homosexual, 2 = other). The HPV status indicator includes the percentage of HPV-positive participants.

### 2.2 Proposed model

#### 2.2.1 Negative binomial model

Here, we suppose that the entry *Y*_*ijg*_ denotes a count value for the *j*^*th*^ OTU of the *i*^*th*^ sample from the *g*^*th*^ batch, with *i* = 1, …, *n*, *j* = 1, …, *m*, *g* = 1, …, *k*. Covariates such as important biomedical, demographic, genomic, and other information based on prior knowledge are represented as *X*_*i*_, which is the vector of fixed effects and β is the vector of the fixed effects coefficients (Zhang et al., 2017).

We describe the negative binomial regression model used for batch effect adjustment. In our approach, it is employed to estimate the count of OTUs, considering the influence of covariates and batch ID as a fixed effect. By treating the batch ID as a fixed effect, this model can directly account for the consistent differences observed across batches. This model specifically targets the non-zero counts of OTUs, denoted as *Y*_*ijg*_ |*Y*_*ijg*_ > 0. The counts are assumed to follow a negative binomial distribution (Tran et al., 2020), represented by *Y*_*ijg*_ |*Y*_*ijg*_ > 0 ~ *NB*(μ_*ijg*_, θ_*jg*_), where μ_*ijg*_ is expected count and θ_*jg*_ is the dispersion parameter of the negative binomial distribution. The equation for this model is expressed as follows (Chen and Li, 2016; Dong et al., 2020; Li et al., 2023; Yirga et al., 2020):


(1) $$\begin{array}{c} log\left(\mu_{ijg}\right)=\sigma_{j}+X_{i}\beta_{j}+\gamma_{jg}+logN_{i} \end{array}$$

In this equation, σ_*j*_ represents the baseline expression level for the *j*^*th*^ OTU, reflecting the unique characteristics of each OTU. **X**_*i*_ is the vector of the covariates for the *i*^*th*^ sample, **β**_*j*_ is the vector of coefficients for the *j*^*th*^ OTU, γ_*jg*_ is the mean batch effect for the *j*^*th*^ OTU in the *g*^*th*^ batch, and log*N*_*i*_ represents the library size, i.e., the total counts across all OTUs in the *i*^*th*^ sample (Johnson et al., 2007; Zhang et al., 2020; Ramakodi, 2021).

The variance of the negative binomial distribution is defined as:


(2) $$\begin{array}{c} Var\left(Y_{ijg}|Y_{ijg}>0\right)=\mu_{ijg}+\theta_{jg}\mu_{ijg}^{2} \end{array}$$

This setup allows us to account for the baseline expression levels, import ant biological covariates, and batch effects in the analysis, providing an adequate structure for modeling OTU counts in microbiome data. To adjust for batch effects, the log-transformed mean parameter log(μ_*ijg*_) is adjusted by subtracting the batch effect term (Van den Berg et al., 2006):


(3) $$\begin{array}{c} log\left(\mu_{ij}^{*}\right)=log\left(\mu_{ijg}\right)-\gamma_{jg} \end{array}$$

where $\mu_{ij}^{*}$ is the adjusted mean parameter. The variance parameter for the *j*^*th*^ OTU in the *g*^*th*^ batch, θ_*jg*_, is averaged across all batches to obtain a consistent variance parameter:


(4) $$\begin{array}{c} \theta_{j}^{*}=\frac{1}{N_{G}}\sum_{g}\theta_{jg} \end{array}$$

where $\theta_{j}^{*}$ is the average variance parameter for the *j*^*th*^ OTU and *N*_*G*_ is the total number of batches. By averaging these parameters, we can apply a consistent variance across all batches, reducing variability caused by individual batch effects that can be caused by non-interest variables (Johnson et al., 2007; Zhang et al., 2020).

The adjusted counts calculated using the refined parameters $\mu_{ij}^{*}$ and $\theta_{j}^{*}$ are modeled to follow negative binomial distribution $Y_{ij}^{*}\sim \;NB\left(\mu_{ij}^{*},\;\theta_{j}^{*}\right)$. Adjusted values $Y_{ij}^{*}$ are then derived by mapping the data from the empirical distribution of the original counts to the batch-free distribution, according to quantile levels. This mapping process ensures that the batch-specific effects are removed, and the adjusted counts are comparable across different batches.

#### 2.2.2 Logistic regression

We proceed with an additional process to address variability at the OTU level using the adjusted OTU count. It initiates with utilizing logistic regression to estimate the probability of nonzero occurrences within the adjusted OTU table (Zhang and Yi, 2020). This probabilistic model serves to identify samples where the presence of OTU is confirmed. Subsequently, quantile regression is applied to these identified samples (Pendegraft et al., 2019).


(5) $$\begin{array}{c} \mathrm{logit}(Pr\;(Y_{ij}^{*}>0))=X_{i}^{T}\zeta +B_{i}^{T}\psi \end{array}$$

(6) $$\begin{array}{c} \mathrm{Pr}\left(\overset{*}{\underset{ij}{Y}}>0\right)=\frac{\exp\left(\overset{T}{\underset{i}{\text{X}}}\zeta +{\text{B}}_{i}^{T}\psi \right)}{1+\exp\left(\overset{T}{\underset{i}{\text{X}}}\zeta +{\text{B}}_{i}^{T}\psi \right)}=q_{ij} \end{array}$$

Here, $Y_{ij}^{*}$ denotes the count of the *j*^*th*^ adjusted OTU in the *i*^*th*^ sample, **X**_**i**_ represents the covariates for this observation, **B**_**i**_ is random effect term for the *i*^*th*^ sample, capturing the batch effect. **ζ** is the coefficient vector linked to covariates, and **ψ** is the coefficient vector associated with batch effects. *q*_*ij*_ denotes the probability of nonzero occurrences within the adjusted OTU table, and it is crucial for understanding the likelihood of observing nonzero counts for a given OTU in the adjusted OTU table.

#### 2.2.3 Composite quantile regression

We employ Composite Quantile Regression (CQR) to robustly model microbiome data, which exhibits zero-inflation and a high frequency of outliers. This method allows for the estimation of conditional values of microbial OTUs across various quantiles. For non-zero microbial counts, the conditional quantile function of the response variable is defined as Ling et al. (2021a,b); Koenker and Hallock (2001):


(7) $$\begin{array}{c} Q_{Y_{ij}^{*}|X_{i},\;{\;Y}_{ij}^{*}>0}\left(\tau \right)=X_{i}^{T}\alpha \left(\tau \right)+B_{i}^{T}\delta \left(\tau \right) \end{array}$$

In this equation, **α**(τ), **δ**(τ) is the vector of regression coefficients specific to quantile level $\tau =\;\left(\frac{1}{k+1},\ldots ,\frac{k}{k+1}\right)$ with a large *k* (e.g., 5^*th*^, 10^*th*^, …, 95^*th*^ *percentiles with k* = 19). This allows us to capture the distributional characteristics of the data across various quantiles. This model diverges from traditional quantile regression by adopting a unified set of regression coefficients across all quantiles. This approach facilitates a more streamlined estimation process that leverages the robustness of quantile regression while simplifying the model complexity typically associated with estimating separate coefficients for each quantile.


(8) $$\begin{array}{c} \sum_{\tau \in T}\sum_{i=1}^{n}\sum_{j=1}^{m}\rho_{\tau }(Y_{ij}^{*}-X_{i}^{T}\alpha \left(\tau \right)-B_{i}^{T}\delta (\tau )) \end{array}$$

In this formula, ρ_τ_(*u*) is the check loss function calculated as ρ_τ_(*u*) = *u*[τ−*I*(*u* < 0)], *T* is the set of target quantiles, *n* is the number of samples, and *m* is the number of OTUs. This formulation reduces the influence of outliers compared to mean regression and provides a deeper understanding of the overall distribution by analyzing data across various quantiles (He et al., 2023).

Then, we incorporate the zero-inflation aspect by accounting for the probability of the response variable being zero. This approach allows us to effectively handle datasets with many zero values by considering the original distribution of the data. We combine the zero probability with the previously estimated quantile function to create a unified model that explains the entire data distribution, including both zero and positive values (Ling, 2019).


(9) $$\begin{array}{c} {\hat{Q}}^{c}\left(\tau_{s}\right)=\{\begin{array}{c} \;0\;,\;\tau <1-q_{ij}\\ \hat{Q}\left(\frac{\tau -\left(1-q_{ij}\right)}{q_{ij}}\right)\;,\;\tau \geq 1-q_{ij} \end{array} \end{array}$$

#### 2.2.4 Reference batch selection

The selection of the reference batch is pivotal in the quantile regression framework as it serves as the baseline to which the distribution of counts (given the OTU is present) are aligned. The reference batch is not chosen based on size or abundance alone, as these factors do not necessarily indicate the quality or representativeness of the batch. To identify potential reference batches that demonstrate homogeneity across multiple microbial datasets, we apply the Kruskal-Wallis test (Kruskal and Wallis, 1952). This non-parametric method assesses whether the median values across different batches are statistically similar, indicating stable baseline conditions across these groups. Batches that do not show significant differences in their medians indicate the possibility of consistent performance across various batches and are shortlisted for further evaluation (Kruskal and Wallis, 1952; Chakraborty et al., 2011). Given the high prevalence of outliers and zero counts in microbiome data, traditional measures of variability such as the standard coefficient of variation (CV) may not provide reliable insights. Instead, we utilize a Robust CV, defined as the ratio of the Median Absolute Deviation (MAD) to the median of the data, multiplied by 100% (Pham-Gia and Hung, 2001):


(10) $$\begin{array}{c} Robust\;CV=\left(\frac{MAD}{Median}\right)*100\% \end{array}$$

This formula offers a more resilient measure against the skewness and anomalies inherent in microbiome data. Among the batches identified as homogeneous by the Kruskal-Wallis test, the batch exhibiting the lowest Robust CV is selected as the optimal reference. This batch is expected to have the least variability, making it the best candidate for minimizing batch effects in the subsequent analyses. Supplementary material including the code and data used in this study, is available at https://github.com/JIWONNP/Composite-Quantile-Regerssion.

## 3 Results

**Figure 1:**
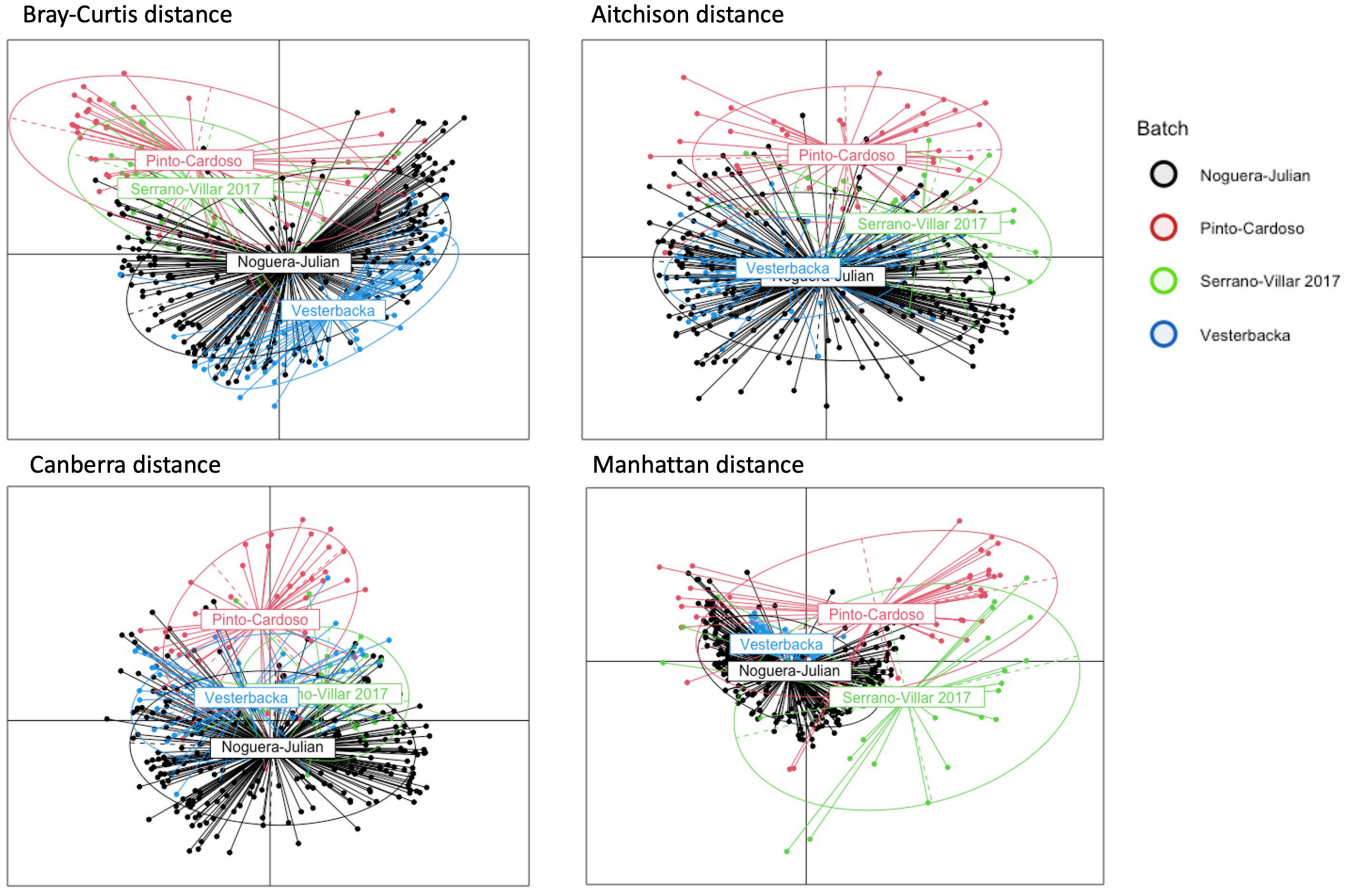
PCoA plots of HIVRC dataset. Each plot corresponds to a different dissimilarity measure. These metrics capture different aspects of the data's structure in pre-correction data. The colors represent batch ID indicating in which of the individual studies that each sample was included. If spatial patterns among data points from various batches are consistent, this is indicative of effective batch effect correction. Distinct colors represent different batches, and the intermingling of these colors rather than clear segregation indicates a high degree of batch effect correction.

**Figure 2:**
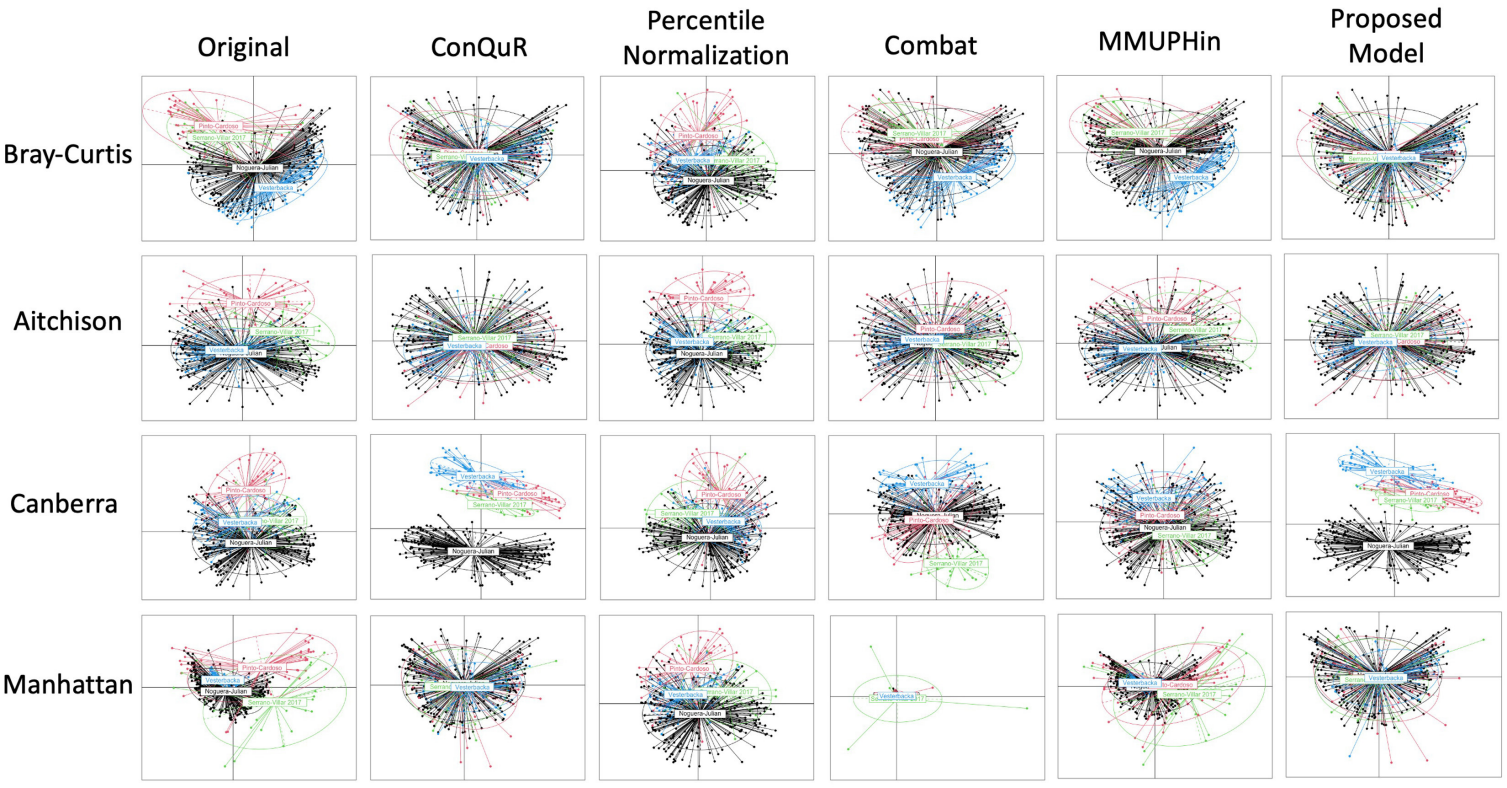
Comparative PCoA plot across diverse methods of HIVRC dataset. Each row shows data points clustered by batch ID. The first column is the original data without any batch effect correction. The following columns show the data after applying existing methods and proposed model. Each row corresponds to a different dissimilarity metric used in the PCoA algorithm. The batch IDs were coded as follows: “Noguera-Julian” = 1, “Pinto-Cardoso” = 2, “Serrano-Villar 2017” = 3, and “Vesterbacka” = 4. The variation across rows demonstrates how the choice of dissimilarity metric affects the visualization of batch effects and their correction.

**Figure 3:**
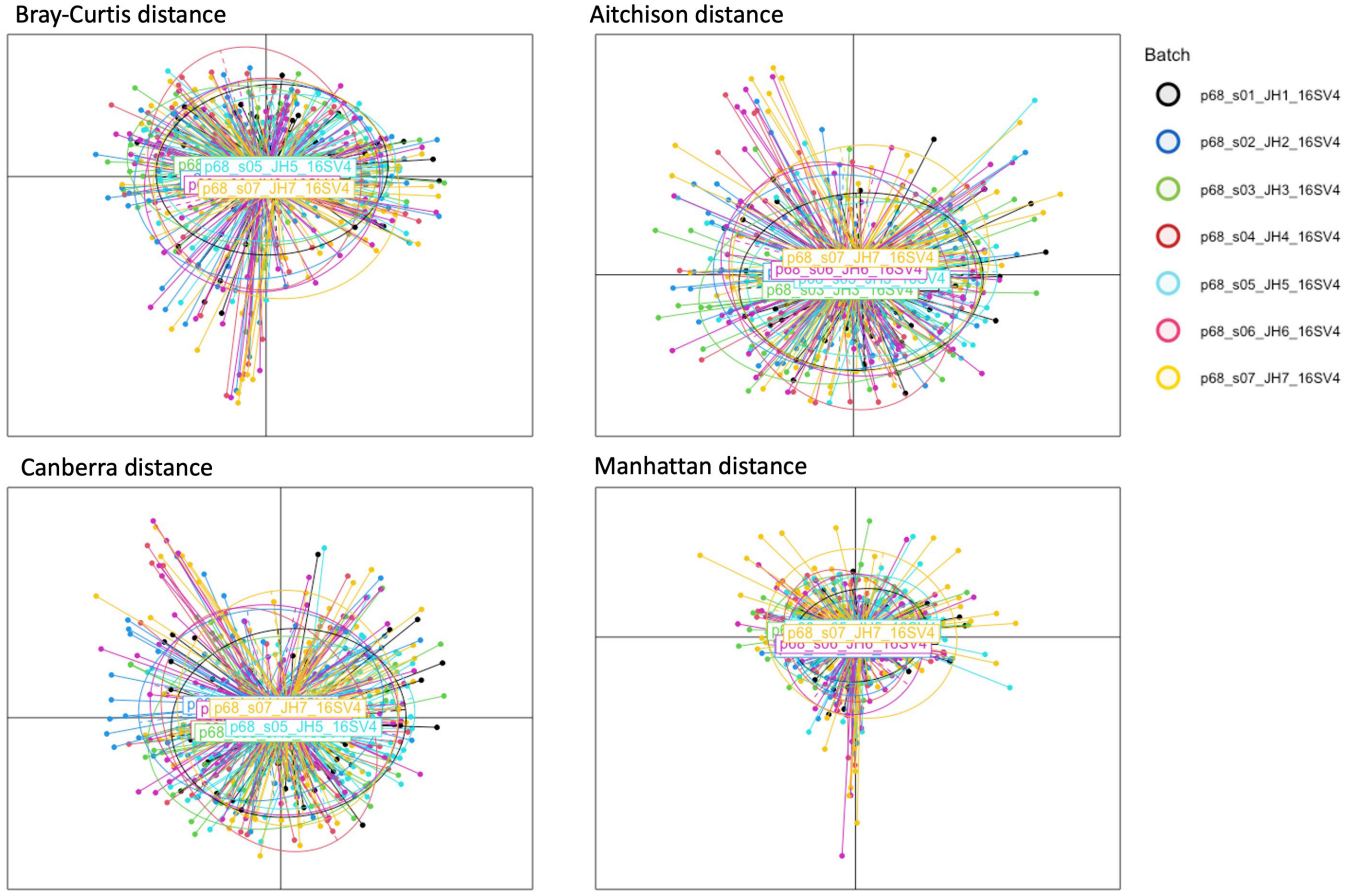
PCoA plots of HPV dataset. Each plot corresponds to a different dissimilarity measure. These metrics capture different aspects of the data's structure. The colors represent seven batch ID indicating individual PlateID that each sample was included.

**Figure 4:**
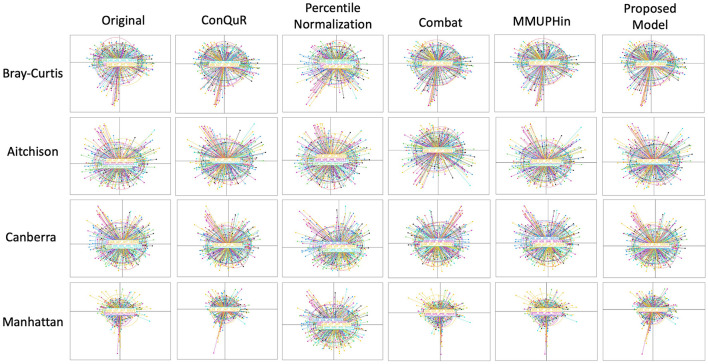
Comparative PCoA plots across diverse methods of HPV dataset. Each row shows data points clustered by 7 batch ID. The first column is the original data without any batch effect correction. The following columns show the data after applying existing methods and proposed model. Each row corresponds to a different dissimilarity metric used in the PCoA algorithm. The batch IDs were coded as follows: “p68_s01_JH1_16SV4” = 1, “p68_s07_JH7_16SV4” = 2, “p68_s02_JH2_16SV4” = 3, and “p68_s03_JH3_16SV4”= 4, “p68_s04_JH4_16SV4” = 5, “p68_s05_JH5_16SV4” = 6, “p68_s06_JH6_16SV4” = 7.

**Figure 5:**
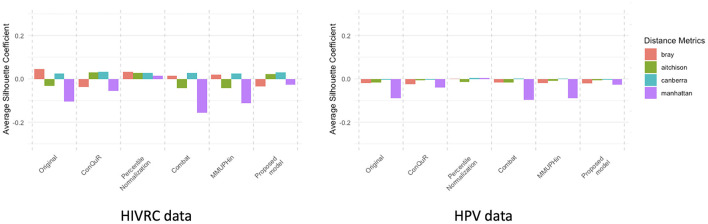
Average Silhouette coefficient of each dataset. The silhouette coefficient measures the quality of clustering (Llet et al., 2004). When the silhouette coefficient is close to 1, it means the samples are well-separated within their own group and distinctly apart from other groups, implying that batch effects are still present. A silhouette coefficient close to 0 suggests that the boundaries between groups are ambiguous and there are no clear distinctions, indicating that the samples are well-mixed and batch effect correction is most ideal. Conversely, a silhouette coefficient close to −1 means that the samples have poor cohesion within their group and are closer to samples in other groups, indicating poor batch effect correction. Four different distance metrics were employed: Bray-curtis (red), Aitchison (green), Canberra (blue), and Manhattan (purple).

In our study, we conducted an empirical evaluation of batch effect correction methods, including MMUPHin, Percentile Normalization, ConQuR, ComBat and our proposed model. To demonstrate the effectiveness of our proposed model, we used PERMANOVA *R*^2^ (Tables 3, 4), Average Silhouette Coefficient for quantification and Principal Coordinates Analysis (PCoA) plots (Figures 1–5) for visualization. A range of dissimilarity metrics commonly used in microbiome research were employed for this evaluation. We compared the values for each method across different dissimilarity metrics (Song et al., 2023): Bray-Curtis, Aitchison, Canberra, and Manhattan. The outcomes of the Kruskal-Wallis test indicate no significant differences between the distributions of batch groups, suggesting that the negative binomial regression model appropriately formed similar distribution pattern by excluding consistent effects across each batch.

**Table 3:** PERMANOVA *R*^2^ of HIVRC dataset.

**Method**	**Distance metric**			
	**Bray-curtis**	**Aitchison**	**Canberra**	**Manhattan**
Original	0.1199^*^	0.0642^*^	0.0808^*^	0.0879^*^
MMUPHin	0.0822^*^	0.0602^*^	**0.0779** ^*^	0.0295^*^
Percentile normalization	0.1191^*^	0.0899^*^	0.1142^*^	0.1136^*^
ConQuR	0.0149^*^	0.0901^*^	0.1628^*^	0.0087^*^
ComBat	0.0637^*^	**0.0564** ^*^	0.0825^*^	0.0137^*^
Proposed model	**0.0128** ^*^	0.0854^*^	0.1493^*^	**0.0065** ^*^

PERMANOVA *R*^2^ represents the proportion of total variance explained by differences between groups (Anderson, 2014; Kelly et al., 2015). In this study, it was utilized to assess whether differences between groups were reduced by comparing the data before and after batch effect correction. A lower *R*^2^ value indicates that the batch effect correction was successfully performed. The values in bold represent the cases where batch correction was most effectively performed for each distance metric. Significant values (*P* ≤ 0.05) are denoted by an asterisk (^*^).

**Table 4:** PERMANOVA *R*^2^ of HPV dataset.

**Method**	**Distance metrics**			
	**Bray-Curtis**	**Aitchison**	**Canberra**	**Manhattan**
Original	0.0379^*^	0.0473^*^	0.0596^*^	0.0291^*^
MMUPHin	0.023^*^	0.0449^*^	0.0677^*^	0.0156^*^
Percentile normalization	0.071^*^	0.039^*^	0.0676^*^	0.0766^*^
ConQuR	0.0154^*^	0.0263^*^	0.0294^*^	0.0032^*^
ComBat	0.0177^*^	0.0422^*^	0.0592^*^	0.0079^*^
Proposed model	**0.002** ^*^	**0.0246** ^*^	**0.0264** ^*^	**0.0029** ^*^

The values in bold represent the cases where batch correction was most effectively performed for each distance metric. Significant values (*P* ≤ 0.05) are denoted by an asterisk (^*^).

Results from the analysis of the Human Immunodeficiency Virus Re-analysis Consortium dataset are presented below. These were analyzed to evaluate the effectiveness of our proposed model in correcting batch effects and maintaining the integrity of the microbiome data. For Bray-Curtis dissimilarity, the proposed model achieved the lowest *R*^2^ value of 0.0128^*^, indicating superior performance in batch effect correction compared to other methods. ConQuR also performed well with a *R*^2^ value of 0.0149^*^, while MMUPHin and ComBat showed moderate performance with *R*^2^ values of 0.0822^*^ and 0.0637^*^, respectively (Table 4). Regarding Aitchison dissimilarity, the proposed model had a higher *R*^2^ value of 0.0854^*^, indicating less effective correction in this specific metric and ComBat demonstrated better performance with R^2^ values of 0.0564^*^. ConQuR showed the highest *R*^2^ value of 0.0901^*^, suggesting the least effective correction in this metric. For Canberra dissimilarity, MMUPHin achieved the lowest *R*^2^ value of 0.0779^*^, closely followed by ComBat with an *R*^2^ value of 0.0825^*^. The proposed model had *R*^2^ value of 0.1493^*^, which was lower than ConQuR's *R*^2^ value of 0.1628^*^, indicating moderate performance. In the case of Manhattan dissimilarity, the proposed model excelled with the lowest *R*^2^ value of 0.0065^*^, demonstrating excellent batch effect correction. ConQuR and ComBat showed moderate performance with *R*^2^ values of 0.0087^*^ and 0.0137^*^, respectively. This analysis confirms the effectiveness of various methods in addressing batch effects, with significant values (*P* ≤ 0.05) marked by an asterisk (^*^). This calculation was conducted using the “adonis” function of vegan package (2.6–6.1) in R.

Overall, our proposed method consistently achieved lower *R*^2^ values, indicating a significant reduction in batch effects across various dissimilarity measures. This performance underscores the robustness and superior effectiveness of our method in correcting batch effects in microbiome data. MMUPHin and Percentile Normalization exhibited intermediate performance across most dissimilarity measures, while ConQuR and ComBat showed varying effectiveness depending on the metric used. The effectiveness of batch effect correction varies across different distance metrics, as each metric considers different aspects of the data. Bray-Curtis and Manhattan metrics reflect compositional differences between samples. The Bray-Curtis metric focuses on the relative abundance of shared OTUs between samples, highlighting how similarly the samples are composed in terms of the OTUs they have in common. Similarly, the Manhattan metric considers the absolute differences in abundance, treating all changes equally. Therefore, Bray-Curtis and Manhattan metrics effectively capture overall abundance differences and compositional changes in data. On the other hand, Aitchison and Canberra metrics emphasize ratio-based differences and small values. The Aitchison metric, used for compositional data, is sensitive to variations in the relative abundance of OTUs, considering ratio fluctuations. The Canberra metric assigns greater weight to changes in small values, highlighting differences in proportions with smaller magnitudes. Due to these characteristics, in composite quantile regression models, the effectiveness of adjustments can be diminished when using distance metrics like Aitchison and Canberra, depending on the selected regression coefficients (Ricotta, 2017). This comprehensive evaluation highlights the proposed model's ability to handle batch effects more effectively, ensuring reliable analysis of microbiome data.

The proposed model consistently achieved the lowest R^2^ values across all distance metrics in the HPV dataset, demonstrating optimal batch effect correction performance with values of 0.002^*^, 0.0246^*^, 0.0264^*^, and 0.0029^*^ for the Bray-Curtis, Aitchison, Canberra, and Manhattan distance metrics, respectively (Table 4). The prominent results of the proposed model with the Bray-Curtis and Manhattan distance metrics can be attributed to the characteristics of the composite quantile regression model described previously.

## 4 Discussion and conclusion

Our model employs a Negative Binomial approach to model the variability within batch effects, aiming to align batch distributions and exclude generalized effects across batches. We select the reference batch through a methodical process, employing the Kruskal-Wallis test and median absolute deviation to identify batches that consistently reflect the general characteristics of the data. Furthermore, our approach incorporates composite quantile regression which addresses the effects within each batch at the OTU level. This procedure ensures that our model not only corrects for obvious systematic differences but also subtly adjusts for less predictable changes within the microbiome data, leading to a more reliable results of biological data across different batches.

When applied to the HIVRC data, and the HPV study data, this model demonstrated considerable success in reducing batch effects. It showed improved correction results compared to the application of conditional quantile regression independently, consistently across these different datasets. By effectively addressing both systematic and non-systematic batch effects, the proposed method ensures a more reliable and accurate analysis of microbiome data. We also verified that similar results were obtained using an additional dataset (Ewha Women's University Medical Center metagenomic urine control data−16S rRNA sequencing data). Given the similarity of observed patterns, we chose to present results from two representative datasets.

Despite the effectiveness of our proposed method, several limitations should be acknowledged. First, microbiome data often contain a high proportion of zeros that can lead to instability in quantile regression at extreme quantiles (e.g., lower 10% and upper 90%) due to the limited number of non-zero data points. This sparsity can affect the robustness and accuracy of the regression estimates at these extreme quantiles. In practice, the dataset with the lowest zero inflation (31%) showed the best results in correcting batch effect based on quantile estimation and based on this, it was determined that the rate of zero inflation could act as a factor that decreases the stability of the model. Furthermore, the performance of the model may vary depending on the metrics or categories of data used. Different datasets or dissimilarity distance measures may influence the effectiveness of the batch effect correction, indicating that the model's performance is not universally consistent across all possible applications. Lastly, the efficacy of our model may be compromised in research designs where the separation of confounding variables from primary variables is challenging. Addressing batch effects in confounded study designs presents considerable difficulties, and careful experimental design can serve as a critical factor in alleviating batch effects. These limitations highlight the need for further refinement of the model to enhance its robustness and applicability across diverse microbiome datasets. Additionally, the model assumes that the count data follow a negative binomial distribution, which may not always be the case in real-world microbiome data. Also, when various trends or patterns clearly emerge across different quantiles of the data, it may be more appropriate to use regression coefficients specialized for each quantile.

## Data Availability

Publicly available datasets were analyzed in this study. This data can be found here: (1) HIVRC [https://www.synapse.org/Synapse:syn18406803/wiki/589668] (ID: syn18406803). (2) HPV [https://www.synapse.org/Synapse:syn26529406/wiki/614952] (ID: syn26529406).
