# Supplementary material for: Composite quantile regression approach to batch effect correction in microbiome data
Source: Front Microbiol. 2025 Feb 25;16:1484183. doi: 10.3389/fmicb.2025.1484183 (PMC11893821; doi:10.3389/fmicb.2025.1484183)
Supplement: Supplementary file 1 [file Data_Sheet_1.pdf]

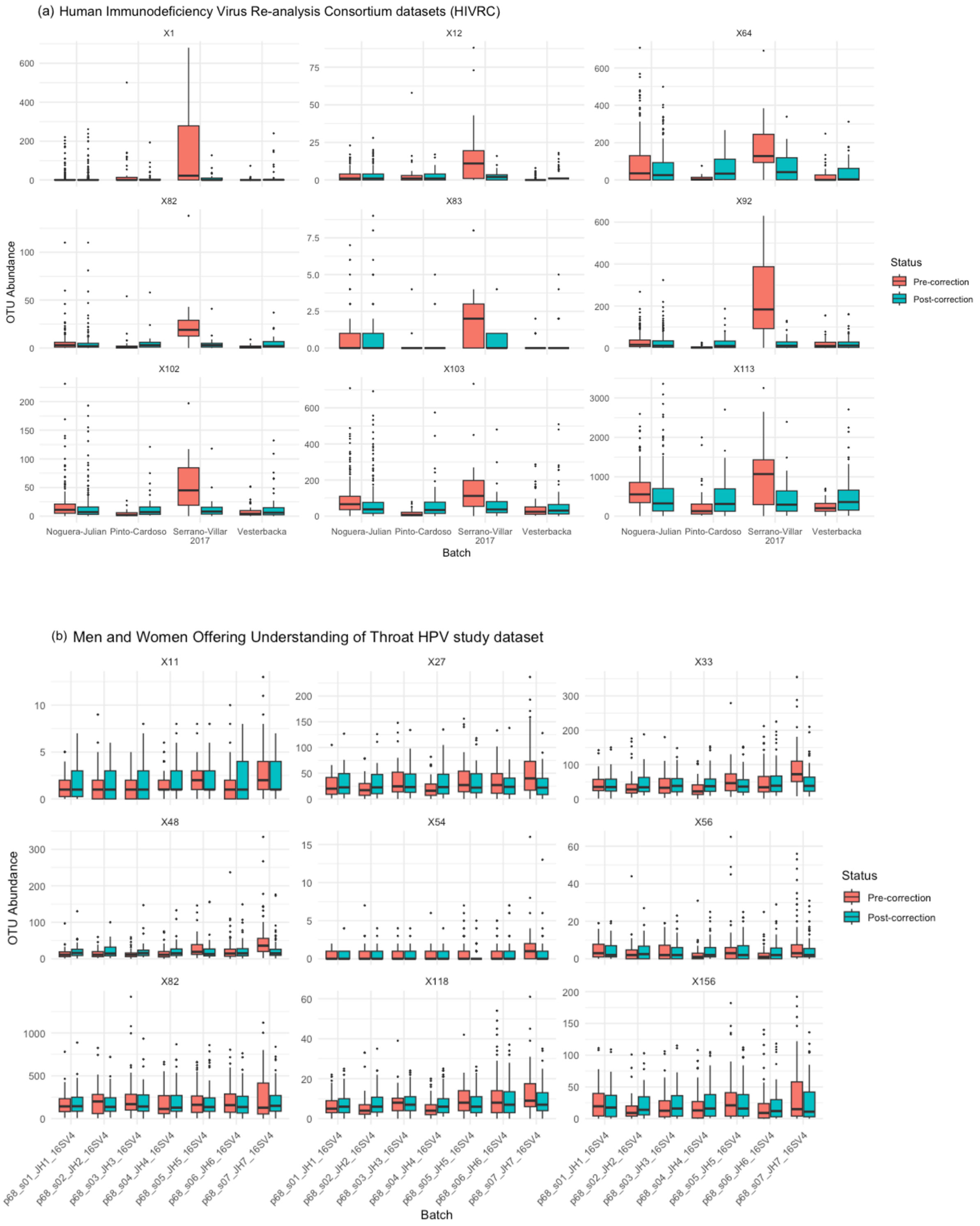

Supplementary Figure 1. Batch-wise OTU Abundance Distribution (Pre vs. Post Correction)
In samples with very low microbial biomass, contaminants present in the reagents used during the experimental process can significantly impact the actual data. We meticulously examined signals that could be identified as contaminants in each dataset based on abnormal expression differences between batches prior to batch effect correction, and we compared the changes observed after the model refined the data. Abnormal expression signals observed in specific batches can be identified as contaminants, and these signals were revealed in each dataset as follows: in the (a) HIVRC dataset, contamination was observed in the third batch, 'Serrano-Villar 2017'; in the (b) HPV dataset, the seventh batch,

'p68_s07_JH7_16SV4', was detected to be contaminated. Specifically, this analysis identified samples where the median expression level in one batch differed from that in another by a factor of 1.5 or more (Wang et al., 2020). The first nine samples meeting this criterion are presented. After applying the batch effect correction, the expression levels of these contaminants were found to closely resemble those of other batches, indicating that the correction process effectively removed residual contamination and ensured proper normalization. (The post-correction results were illustrated using the values adjusted by the proposed model.)
